# Relationship between Hexokinase and the Aquaporin PIP1 in the Regulation of Photosynthesis and Plant Growth

**DOI:** 10.1371/journal.pone.0087888

**Published:** 2014-02-03

**Authors:** Gilor Kelly, Nir Sade, Ziv Attia, Francesca Secchi, Maciej Zwieniecki, N. Michele Holbrook, Asher Levi, Victor Alchanatis, Menachem Moshelion, David Granot

**Affiliations:** 1 Institute of Plant Sciences, Agricultural Research Organization, The Volcani Center, Bet Dagan, Israel; 2 The Institute of Plant Sciences and Genetics in Agriculture, The Robert H. Smith Faculty of Agriculture, Food and Environment, The Hebrew University of Jerusalem, Rehovot, Israel; 3 Plant and Environmental Sciences, University of California Davis, Davis, California, United States of America; 4 Organismic and Evolutionary Biology, Harvard University, Cambridge, Massachusetts, United States of America; 5 Institute of Agricultural Engineering, Agricultural Research Organization, The Volcani Center, Bet Dagan, Israel; University of Nottingham, United Kingdom

## Abstract

Increased expression of the aquaporin *NtAQP1*, which is known to function as a plasmalemma channel for CO_2_ and water, increases the rate of both photosynthesis and transpiration. In contrast, increased expression of Arabidopsis hexokinase1 (*AtHXK1*), a dual-function enzyme that mediates sugar sensing, decreases the expression of photosynthetic genes and the rate of transpiration and inhibits growth. Here, we show that *AtHXK1* also decreases root and stem hydraulic conductivity and leaf mesophyll CO_2_ conductance (*g*_m_). Due to their opposite effects on plant development and physiology, we examined the relationship between *NtAQP1* and *AtHXK1* at the whole-plant level using transgenic tomato plants expressing both genes simultaneously. *NtAQP1* significantly improved growth and increased the transpiration rates of *AtHXK1*-expressing plants. Reciprocal grafting experiments indicated that this complementation occurs when both genes are expressed simultaneously in the shoot. Yet, *NtAQP1* had only a marginal effect on the hydraulic conductivity of the double-transgenic plants, suggesting that the complementary effect of *NtAQP1* is unrelated to shoot water transport. Rather, *NtAQP1* significantly increased leaf mesophyll CO_2_ conductance and enhanced the rate of photosynthesis, suggesting that *NtAQP1* facilitated the growth of the double-transgenic plants by enhancing mesophyll conductance of CO_2_.

## Introduction

Aquaporins (AQPs), also known as MIPs (major intrinsic proteins), are integral membrane proteins that increase the permeability of membranes to water, as well as small uncharged molecules [1]. Of all kingdoms, the plant kingdom contains the largest known AQP family consisting over 30 members [2], [3]. There are 35 AQPs in Arabidopsis (*Arabidopsis thaliana* [4]), 36 in maize (*Zea mays* [1]) and 37 in tomato (*Solanum lycopersicum* [5]. Based on sequence similarities, AQPs have been divided into five subgroups: plasma membrane intrinsic proteins (PIPs), tonoplast intrinsic proteins (TIPs), NOD26-like intrinsic proteins (NIPs), small basic intrinsic proteins (SIPs) and X intrinsic proteins (XIP) [4], [6]. Plant PIPs can be divided into two major groups, PIP1 and PIP2, on the basis of their sequences and water-channel activity. PIP2 proteins exhibit high levels of water-channel activity in *Xenopus* oocytes and yeast vesicles; whereas PIP1 proteins often have relatively low permeability to water [7]–[12].

Evidence for the role of PIP1 aquaporin *in planta* has come from mutant analyses and the manipulation of PIP1 expression in plants. Analysis of Arabidopsis mutants has shown that *AtPIP1*,*2* can account for a significant portion of aquaporin-mediated leaf water transport [13]. The antisense expression of *AtPIP1,2* in Arabidopsis has been associated with reductions in the membrane hydraulic conductivity of isolated protoplasts and decreased total root hydraulic conductivity [14], [15]. Antisense suppression of *NtAQP1* (a member of the PIP1 subgroup) in tobacco (*Nicotiana tabacum*) lowered the level of expression of several PIP1 homologues and resulted in a significant decrease in protoplast membrane water permeability, reduced root hydraulic conductivity and decreased transpiration [16],[17].

The results of heterologous expression in *Xenopu*s oocytes suggest that, in addition to functioning as a water channel, *NtAQP1* is also a membrane CO_2_ pore that facilitates the transport of CO_2_ across membranes [7], [18]. The movement of CO_2_ between the substomatal cavities and the sites of carboxylation within chloroplasts, through plasma and chloroplast membranes, is generally termed leaf mesophyll conductance (*g*_m_) [19]. The ability of *NtAQP1* and its Arabidopsis homolog *AtPIP1,2* to function as CO_2_ membrane transport facilitators has been demonstrated in *in vivo* experiments. Increased expression of *NtAQP1* in tobacco plants enhanced CO_2_ incorporation and stomatal conductance; whereas antisense suppression of *NtAQP1* had the opposite effect [18]. In other studies, overexpression of *AtPIP1,2* or *NtAQP1* in tobacco plants significantly enhanced the rates of growth, transpiration and photosynthesis [20]–[22]; whereas antisense suppression of *NtAQP1* in tobacco plants and T-DNA insertion Arabidopsis mutants in *AtPIP1,2* reduced *g*_m_ and led to lower rates of photosynthesis [21], [23], [24].

Unlike *NtAQP1*, overexpression of Arabidopsis hexokinase (*AtHXK1*) in Arabidopsis and tomato plants decreased photosynthesis, transpiration and growth [25], [26]. AtHXK1 is a sugar-sensing enzyme that monitors glucose levels, most likely in mesophyll cells of photosynthetic tissues. When glucose levels are sufficiently high, this enzyme inhibits the expression of photosynthetic genes, decreases chlorophyll levels and reduces the rate of photosynthesis [25]–[29]. In addition, AtHXK1 also stimulates stomatal closure and decreases transpiration in response to increasing sugar levels [26], [30]. In light of the opposite effects of *AtHXK1* and *NtAQP1* on photosynthesis and growth, we examined the relationship between *AtHXK1* and *NtAQP1* using double-transgenic plants that express *AtHXK1* and *NtAQP1* simultaneously. We found that *NtAQP1* significantly compensated for the growth inhibition imposed by *AtHXK1*, primarily by enhancing mesophyll CO_2_ conductance and the rate of photosynthesis, while the hydraulic conductivity in those plants remained unchanged.

## Materials and Methods

### Construction of transgenic AQP1 plants

Cloning of the full-length cDNA of the tobacco (*Nicotiana tabacum*) *NtAQP1* under the control of the 35S constitutive promoter was performed as described in [22]. MP-1 lines (*Solanum lycopersicum* cv. MP-1) were transformed using the *Agrobacterium tumefaciens* transformation method [31]. Plants were assayed for the presence of *NtAQP1* by PCR using the following primers: 35Sprom-Fwd: TATCCTTCGCAAGACCCTCC, and NtAQP1-Rev: TGCCTGGTCTGTGTTGTAGAT.

### Plant material

All experiments were conducted using wild-type (WT) tomato (*Solanum lycopersicum* cv. MP-1), isogenic independent transgenic homozygote tomato lines expressing different levels of the Arabidopsis *AtHXK1* (HK37, HK4 and HK38 lines), as previously described in Dai [25], and an isogenic *NtAQP1*-expressing transgenic homozygote line AQP1. Double-transgenic homozygous plants *NtAQP1xAtHXK1* (AQP1xHK4) were generated by crossing the AQP1 and HK4 parental lines. After self-pollination of the F1 hybrid plants, screening for F2 plants homozygous for both genes was performed using the highly sensitive Taq-Man DNA quantitative PCR method with specific probes, as described by German et al. [32]. Further validation of homozygosity was carried out by PCR analysis of tens of F3 plants using specific primers for *NtAQP1* (35Sprom-Fwd-TATCCTTCGCAAGACCCTCC, *NtAQP1*-Rev- TGCCTGGTCTGTGTTGTAGAT) and *AtHXK1* (Fwd-CGGGAAGCAAGAGCGTGTT, Rev-CTCCTCGGGTTGCTATGATG).

### Measurements of root hydraulic conductance

The hydraulic conductance of the tomato root system (*L*_r_) was assessed using plants grown hydroponically and was determined by measuring the flow induced in response to 1 bar of applied pressure. De-topped root systems were fitted with a plastic tube filled with deionized water and connected to a beaker located on a balance (Sartorius ±0.01 mg). The root system was sealed in a chamber containing the hydroponic solution in which the plants had been grown. The pressure in the chamber was regulated using a needle valve, which was adjusted to allow a small leak into the chamber, so that the air used to pressurize the chamber also served to aerate the medium. Water flow through the root system was automatically recorded by a computer at 30 s intervals. At the end of each experiment, the roots were dried in an oven for 72 h at 90°C and the dry weight of the root system was then measured.

### Measurements of stem hydraulic conductivity

Stem hydraulic conductivity was assessed on five to seven stems of each genotype. Short sections of stems (∼2–3 cm long) were cut under water directly from the intact plants to prevent embolisms caused by air entering into the cut vessels. Stems were connected to a balance (Sartorius ±0.1 mg) by a plastic tube and a filtered 10 mM KCl solution, used as a perfusion solution, was located on the balance in a beaker. Stem segments were first perfused under elevated pressure (0.2 MPa) to remove any embolisms and hydraulic conductivity (*K*_s_) was then calculated as the flow rate multiplied by the length of the stem segment and divided by the pressure gradient.

Xylem cross-sectional area was microscopically determined for each stem to allow the calculation of the xylem-specific stem conductivity (*K*_sx_, which equals *K*_s_ divided by total xylem area). Free-hand cross-sections were excised and stained for a few seconds in a diluted Safranin solution. The sections were then rinsed in deionized water for few minutes and photographed under a compound microscope. Xylem area was later determined using the ImageJ software (http://rsbweb.nih.gov/ij/).

### Measurements of whole-plant transpiration

Whole-plant transpiration rates and relative daily transpiration (RDT) were determined using lysimeters, as described in detail by Sade et al. [22]. WT, AQP1, HK4, AQP1xHK4 and grafted plants were planted in 3.9-L pots and grown under controlled conditions. Each pot was placed on a temperature-compensated load cell with digital output and was sealed to prevent evaporation from the surface of the growth medium. A wet, vertical wick made of 0.14 m^2^ cotton fibers partially submerged in a 1-L water tank was placed on a similar load cell and used as a reference for the temporal variations in the potential transpiration rate. The output of the load cells was monitored every 10 s and the average readings over 3-min intervals were logged in a data logger for further analysis. The whole-plant transpiration rate was calculated as a numerical derivative of the load cell output following a data-smoothing process [22]. The plant's daily transpiration rate was normalized to the total leaf area [measured using a LI-COR area meter, model Li-3100; (Lincoln, Nebraska, USA)] or to total plant weight, and to the data for neighboring submerged wick. These figures were averaged for each line and graft type (amount taken up by the wick daily = 100%).

### Protein extraction and analysis of hexokinase activity

Protein extraction and hexokinase activity measurements were performed as described by Dai et al. [25].

### RNA extraction, cDNA preparation and quantitative real-time PCR

Leaf tissue was harvested from WT, AQP1, HK4 and AQP1xHK4 plants and total RNA was extracted from that tissue using EZ-RNA kit (Biological Industries Co., Beit Haemek, Israel) according to the manufacturer's protocol. The RNA was treated with DNase (Ambion, Austin, TX, USA), according to the manufacturer's instructions, to degrade any residual DNA. The presence of RNA was confirmed by gel electrophoresis and DNA degradation was confirmed by PCR. For cDNA preparation, total RNA (1 µg) was taken for reverse transcription-PCR using MMLV RT (ProMega, Madison, WI, USA) in a 25-µl reaction with 2 µl of random primers (ProMega, Madison, WI, USA) and 1 µl of mixed poly-dT primers. cDNA samples were diluted 1∶7 in RNase-free- DEPC (Diethylpyrocarbonate) water. Quantitative real-time PCR reactions were performed using SYBR Green mix (Thermo-Scientific, Waltham, Massachusetts, USA). Reactions were run in a RotorGene 6000 cycler (Corbett, Mortlake, New South Wales, Australia). Following an initial pre-heating step at 95°C for 15 min, there were 40 cycles of amplification consisting of 10 s at 95°C, 15 s at 55°C, 10 s at 60°C and 20 s at 72°C. Results were analyzed using RotorGene software. Data were normalized using *SlCyP* (cyclophilin – accession; M55019) as a reference gene. The following primers were used for amplification: *SlCAB1* (Fwd-TTGTGTTGATGGGAGCCGT, Rev-AAGGCCTAATGGGTCGAAGCT), *SlCyP* (Fwd-CGTCGTGTTTGGACAAGTTG, Rev-CCGCAGTCAGCAATAACCA) and TRAMP (Fwd-GTGAAGGGCTTCATGGTAGG, Rev-GGAAGTGGTGCCAAAATAGG). For each line tested, five to six independent samples were examined, with two replicates per sample.

### Gas-exchange measurements and estimation of *g*_m_ based on gas exchange and chlorophyll fluorescence

Gas exchange was measured using a Li-6400 portable gas-exchange system (LI-COR, Lincoln, Nebraska, USA). Analysis was performed on fully expanded leaves (5^th^–6^th^ leaf from top) of plants growing under favorable conditions. All measurements were conducted between 10:00 AM and 1:00 PM. Photosynthesis was induced in saturating light (1200 µmol m^−2^ s^−1^) with 370 µmol mol^−1^ CO_2_ surrounding the leaf (*C*_a_) and 15% photosynthetically active photon flux density. The flow rate was set to 500 µmol air s^−1^. The leaf-to-air vapor pressure deficit was kept around 1-2.5 kPa during all measurements. Leaf temperature was ∼28°C (ambient temperature).

Chlorophyll fluorescence was measured using the LI-6400 open gas-exchange system with an integrated fluorescence chamber head (LI-6400-40; LI-COR). The actual photochemical efficiency of photosystem II (*Φ*_PSII_) was calculated using Equation 1. Steady-state fluorescence (*F*_s_) and maximum fluorescence were measured during a light-saturating pulse of ca. 8000 µmol m^−2^ s^−1^ (*F*_m_′), following the protocol used by Genty [33]. This procedure was repeated four times with similar results.
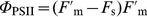
(1)


The electron transport rate (*J*) was then calculated using Equation 2, in which PPFD is the photosynthetically active photon flux density, *α* is leaf absorbance and *β* reflects the partitioning of absorbed quanta between photosystem II and photosystem I (PSII and PSI). Leaf absorbance (*α*) was measured between wavelengths of 400–700 nm, using an integrated sphere device (LI-COR, 1800–12s), as described by [34]–[36].There were six to eight independent biological repeats for each line. A *β* value of 0.5 was used as described in [37]–[40].
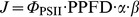
(2)


From combined gas-exchange and chlorophyll-fluorescence measurements, the mesophyll conductance for CO_2_ (*g*_m_) was estimated as *g*_m_ = *A*_N_/(*C*_i_−(Γ* (*J*+8·(*A*_N_+R_l_)))/(*J*−4·(*A*_N_+R_d_))), where *A*_N_ and *C*_i_ were obtained from gas-exchange measurements, as described by [41]. A value of 49.2 µmol mol^−1^ for the CO_2_ compensation point under non-respiratory conditions (Γ*) was used, after [42]. Respiration in the light (R_l_) was determined from dark respiration values (R_d_) obtained with the Li-6400 instrument at 25°C (−1.4±0.2 µmol CO_2_ m^−2^ s^−1^). A value equal to half of the dark respiration was used as a surrogate for R_l_ [43].

## Results

### *AtHXK1* decreases root and stem hydraulic conductivity

**Figure 1:**
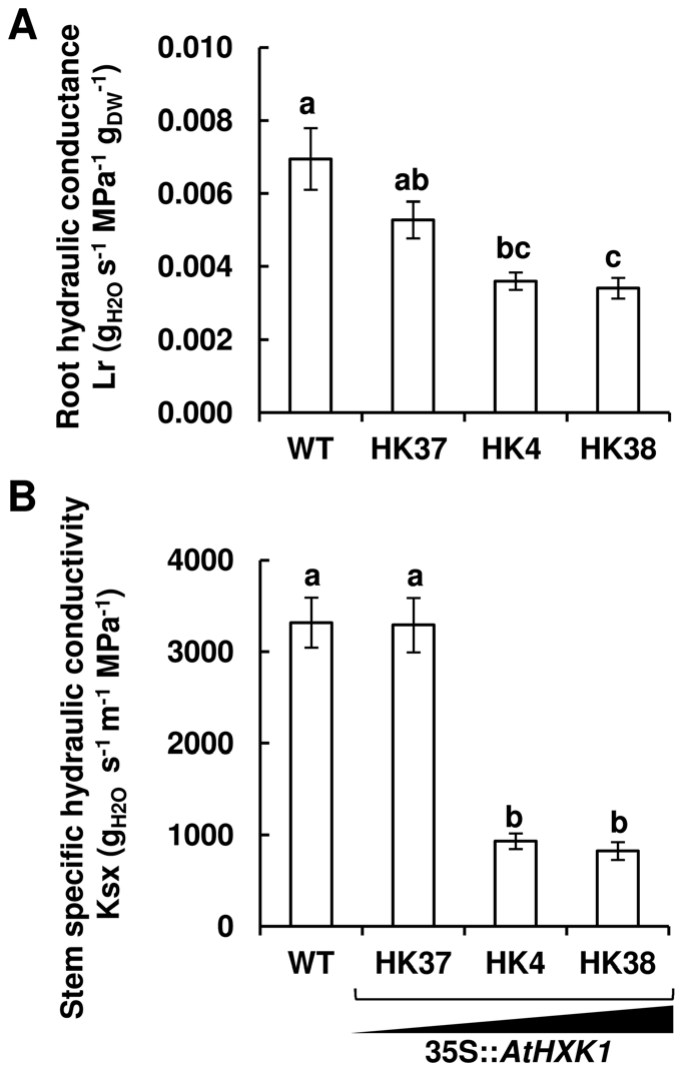
*AtHXK1* decreases root hydraulic conductance and stem hydraulic conductivity. Root conductance (A) and xylem-specific stem hydraulic conductivity (B) were determined for control (WT) and transgenic plants expressing different levels of *AtHXK1* (*AtHXK1* expression from moderate to high: HK37<HK4<HK38; [25]). Data are means ± SE (*n*≥6 for *L*_r_; *n*≥5 for *K*_sx_). Different letters indicate a significant difference (*t* test, *P*<0.05).

To examine the effects of *AtHXK1* on hydraulic proprieties, we measured the root conductance and stem hydraulic conductivity of tomato lines expressing elevated levels of *AtHXK1* (Fig. 1). HK37, HK4 and HK38 are very well characterized independent isogenic transgenic tomato lines that express *AtHXK1* at different levels [25]. These lines exhibit HXK activity that is about 2, 5 and 6 times higher than that of WT plants, respectively [25]. The root hydraulic conductance (*L*_r_) and xylem-specific stem hydraulic conductivity (*K*_sx_) of HK4 and HK38 lines with high levels of *AtHXK1* expression were significantly lower than those of WT plants (Fig. 1A and 1B).

### *NtAQP1* complements *AtHXK1*-mediated growth inhibition

While *AtHXK1* decreases hydraulic conductivity, photosynthesis and growth [25], [26], *NtAQP1* increases hydraulic conductivity and enhances photosynthesis and growth [20], [22]. In light of these opposite effects of *AtHXK1* and *NtAQP1*, we were interested in exploring the relationship between *NtAQP1* and *AtHXK1* at the whole-plant level. To that end, we developed tomato line expressing *NtAQP1* against the same genetic background (MP1 [31]) as that of the HK lines and assigned it AQP1. Expression of the *NtAQP1*gene and the level of NtAQP1 protein were determined by quantitative PCR and Western blot analysis, respectively (Fig. S1). We then created double-transgenic plants expressing both *AtHXK1* and *NtAQP1* simultaneously by crossing AQP1 lines with the HK4 line. Plants homozygous for both genes were identified and are referred to as AQP1xHK4.

**Figure 2:**
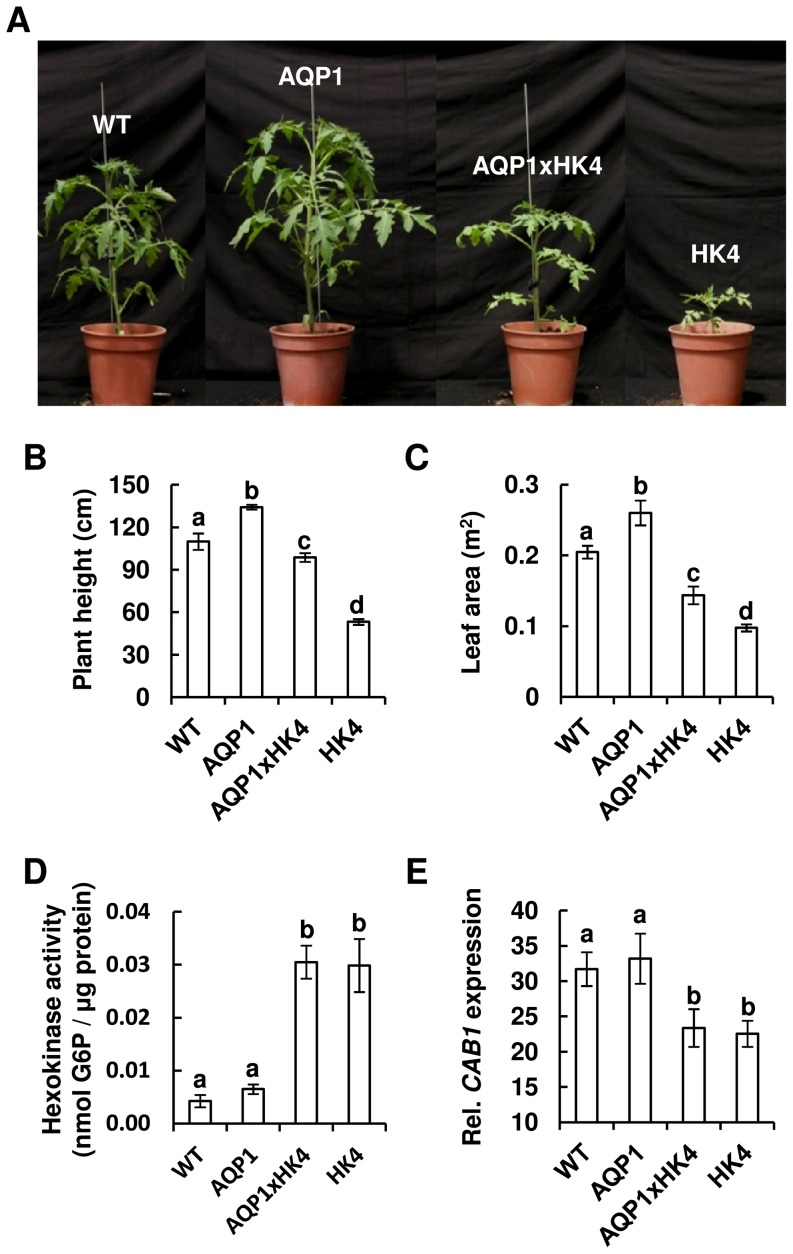
*NtAQP1* complements growth inhibition of *AtHXK1*. (A) Representative images of 5-week-old tomato plants homozygous for *NtAQP1* (AQP1), *AtHXK1* (HK4) or both genes (AQP1xHK4). (B) Height (*n*≥8) and (C) leaf area (*n*≥6) of 9-week-old plants. (D) Hexokinase activity was determined using protein extracted from mature leaves of WT, AQP1, HK4 and AQP1xHK4 plants. Data are means of five independent biological repeats ± SE. (E) Relative expression of *SlCAB1* (*Solanum lycopersicum* a/b binding protein) in WT, AQP1, HK4 and AQP1xHK4 plants. Data are means of five-six independent biological repeats ± SE. (B–E) Different letters indicate a significant difference (*t* test, *P*<0.05).

AQP1xHK4 plants were taller and had more leaf area than the HK4 parent line (Fig. 2), suggesting that *NtAQP1* complemented the growth-inhibition effects of *AtHXK1*. To verify that this complementation effect was not the result of lowered expression of *AtHXK1*, HXK activity and the sugar-sensing effects of HXK were checked. HXK activity in the double-transgenic plants was similar to that of the HK4 parent plants, about 7-fold higher than that of the control WT and the AQP1 (homozygote) parent plants (Fig. 2D). We also examined the effect of HXK on the expression of the well-established sugar-sensing photosynthesis marker gene *CAB1*, which is known to be repressed by *AtHXK1* [26], [27], [29]. *CAB1* expression in AQP1xHK4 was repressed to levels similar to those observed in the HK4 plants (Fig. 2E), indicating that *AtHXK1* mediated sugar-sensing effects in the double-transgenic plants. These results suggest that the growth complementation effects of *NtAQP1* do not stem from suppression of HXK activity, but rather are probably due to epistatic physiological effects of *NtAQP1*.

### Growth complementation of AQP1xHXK is related to *NtAQP1* copy number

**Figure 3:**
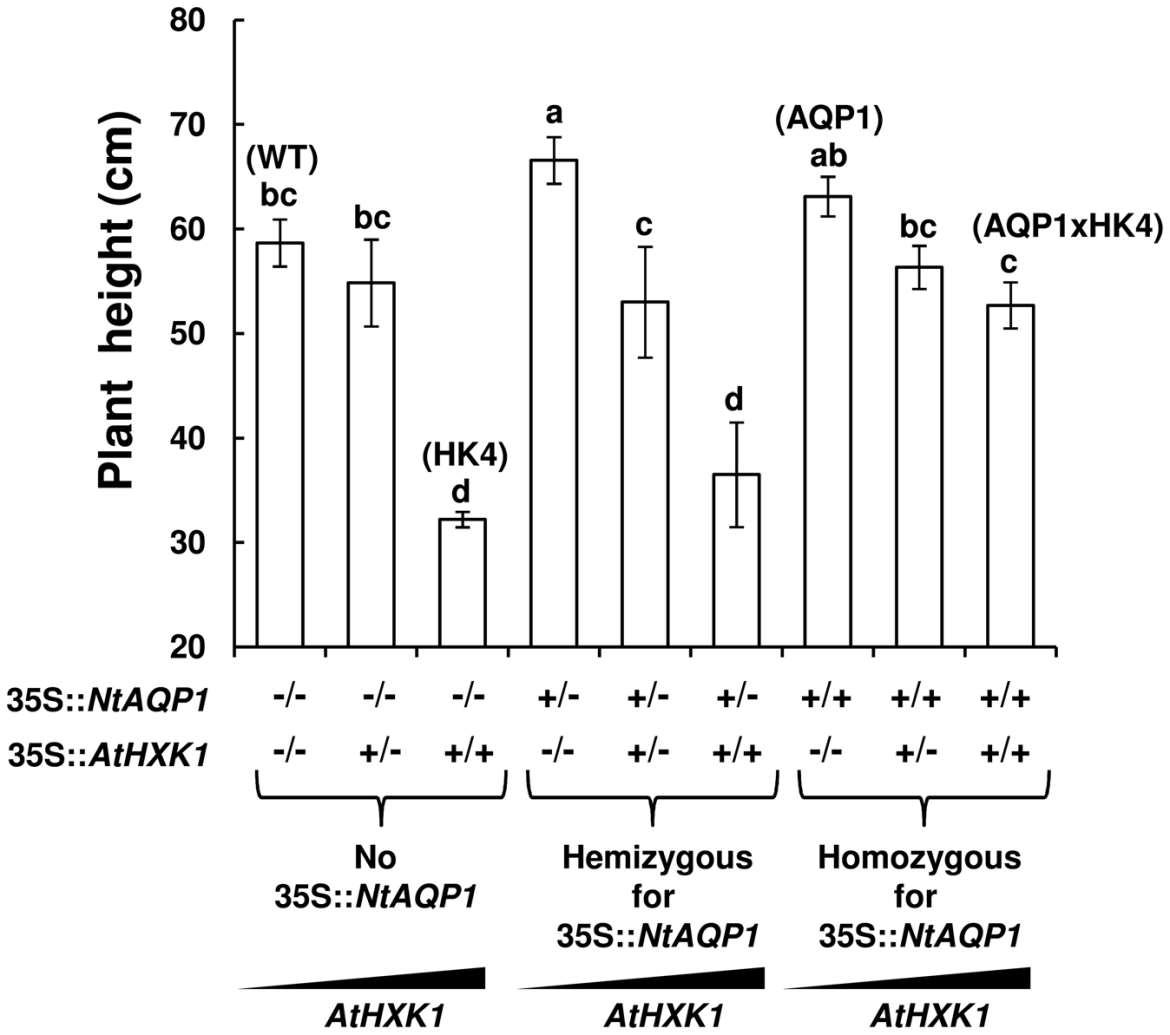
Plant growth is affected by *NtAQP1* and *AtHXK1* gene dosage. Height of transgenic plants with one copy (hemizygous, +/−) or two copies (homozygous, +/+) of *AtHXK1* and *NtAQP1*. The zygosity state of each gene is specified on the x-axis. −/− indicates the absence of the specified gene, −/+ indicates hemizygosity and +/+ indicates homozygosity. Three left columns: no *NtAQP1*; middle three columns: hemizygous for *NtAQP1*; three right columns: homozygous for *NtAQP1*. Data are means of at least six independent repeats ± SE. Different letters indicate a significant difference (*t* test, *P*<0.05).

The epistatic effects of *NtAQP1* on plant growth were observed primarily in plants homozygous for both genes, *NtAQP1* and *AtHXK1* (Fig. 3). Crossing AQP1xHK4 with WT, HK4 or AQP1 lines yielded plants that were heterozygous or homozygous for *NtAQP1*, *AtHXK1* or both genes. Only plants that were homozygous for *AtHXK1* exhibited significant growth inhibition, and *NtAQP1* enhanced the growth of *AtHXK1* homozygous plants only when present in the homozygous state (Fig. 3). Plants that were heterozygous for *NtAQP1* and lacked *AtHXK1* displayed slightly improved growth, but that effect was abolished in the presence of one or two copies of *AtHXK1*, suggesting a dosage effect in the relationship between *NtAQP1* and *AtHXK1*.

### *NtAQP1* enhances the stomatal conductance and transpiration of *AtHXK1* plants

**Figure 4:**
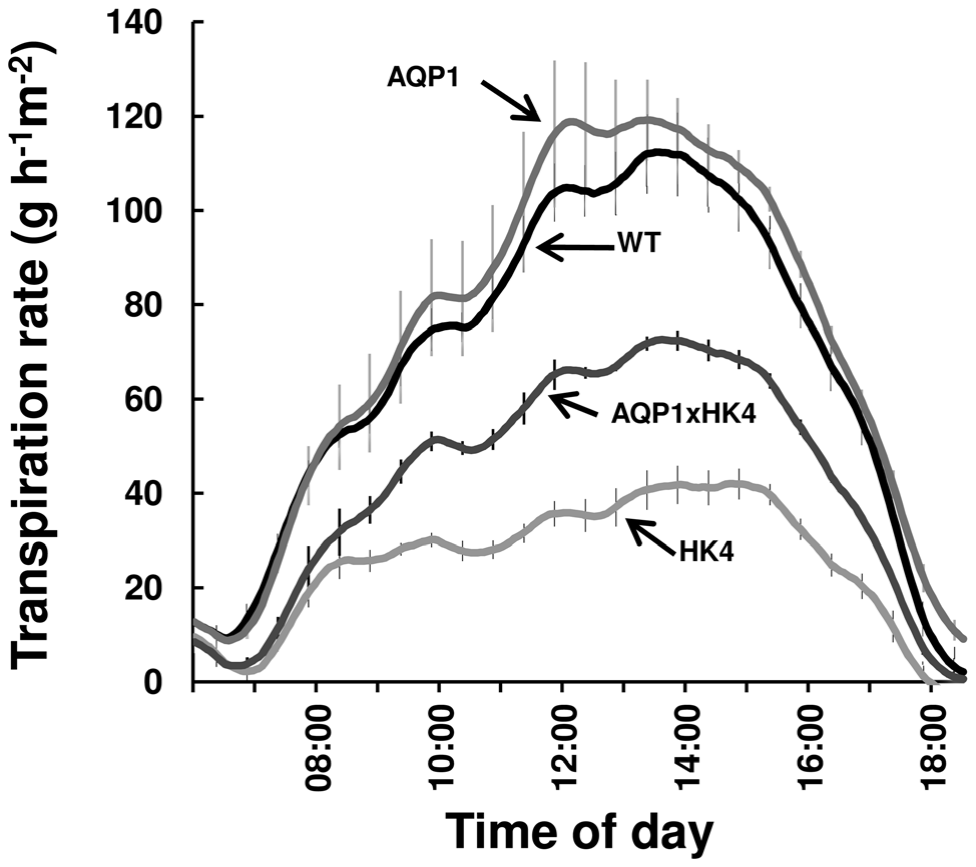
Transpiration rate of AQP1xHK4 plants. The rate of transpiration was monitored continuously throughout the day for each line (AQP1, HK4, AQP1xHK4 and WT). The presented data are the means ± SE for each 10^th^ sampling point (*n* = 6). The transpiration data were normalized to the total leaf area and the amount taken up by the neighboring submerged fixed-size wick each day, which was set to 100%.

In previous studies, overexpression of *AtHXK1* decreased stomatal conductance and transpiration; whereas overexpression of *NtAQP1* increased stomatal conductance and transpiration [18], [20]–[22], [26], [30]. Therefore, we tested the combined effects of *AtHXK1* and *NtAQP1* on stomatal conductance and transpiration. Stomatal conductance (*g_s_*) of HK4 plants was significantly lower than that of WT plants (Table 1; [30]). Meanwhile, the *g_s_* of the double-transgenic plants was similar to that of the WT plants (Table 1). Continuous measurements of whole-plant transpiration per unit leaf area over the course of the day revealed significantly lower transpiration rates in HK4 plants, as compared to WT and AQP1 plants (Fig. 4). Yet, the double-transgenic plants had intermediate-level transpiration rates that were higher than those of HK4 plants (Fig. 4). These results indicate that *NtAQP1* enhanced stomatal conductance and compensated for the limitations imposed on transpiration rates by *AtHXK1*.

**Table 1:** Photosynthetic and hydraulic characteristics of WT, AQP1, AQP1xHK4 and HK4 plants.

	WT	AQP1	AQP1xHK4	HK4
***L*** **_r_ (g_H2O_ s^−1^ MPa^−1^ g_DW_^−1^)**	**0.00319±0.0003 (7) a**	**0.00269±0.0005 (7) ab**	**0.00201±0.0004 (10) b**	**0.00178±0.00008 (6) b**
***K*** **_sx_ (g_H2O_ s^−1^ m^−1^ MPa^−1^)**	**1501.48±167.7 (7) a**	**1081.73±196.2 (7) a**	**381.61±36.6 (5) b**	**274.25±16.8 (6) b**
***A*** **_N_ (µmol CO_2_ m^−2^ s^−1^)**	**28.189±0.68 (18) a**	**27.580±0.60 (20) a**	**28.073±0.49 (15) a**	**20.831±1.34 (13) b**
***g*** **_s_ (mol H_2_O m^−2^ s^−1^)**	**0.702±0.04 (18) a**	**0.637±0.04 (20) ab**	**0.697±0.03 (15) a**	**0.525±0.06 (13) b**
***g*** **_m_ (mol CO_2_ m^−2^ s^−1^)**	**0.248±0.019 (18) a**	**0.232±0.014 (20) ab**	**0.2004±0.007 (15) b**	**0.148±0.015 (13) c**
***C*** **_i_ (µmol CO_2_ mol^−1^)**	**312.5±2.67 (18) a**	**305.1±4.39 (20) a**	**313.27±2.39 (15) a**	**311.29±5.53 (13) a**
***C*** **_c_ (µmol CO_2_ mol^−1^)**	**185.6±6.27 (18) a**	**183.8±6.34 (20) a**	**173.9±3.09 (15) a**	**154.9±6.26 (13) b**
***J*** ** (µmol m^−2^ s^−1^)**	**233.3±3.19 (18) b**	**238.8±3.5 (20) ab**	**247.7±2.16 (15) a**	**204.05±6.22 (13) c**

*L*_r_, root hydraulic conductance; *K*_sx_, xylem-specific stem hydraulic conductivity; *A*_N_, net photosynthesis; *g*_s_, stomatal conductance; *g*_m_, mesophyll CO_2_ conductance; *C*_i_, substomatal CO_2_ concentration; C_c_, Chloroplast CO_2_ concentration; *J*, the rate of electron transport. Presented data are means ± SE (*n*, number of replicates, as indicated in parentheses). Different letters in a row indicate significant differences (*t* test, *P*<0.05).

### Growth and transpiration complementation occurs when *NtAQP1* and *AtHXK1* are simultaneously expressed in the shoot

**Figure 5:**
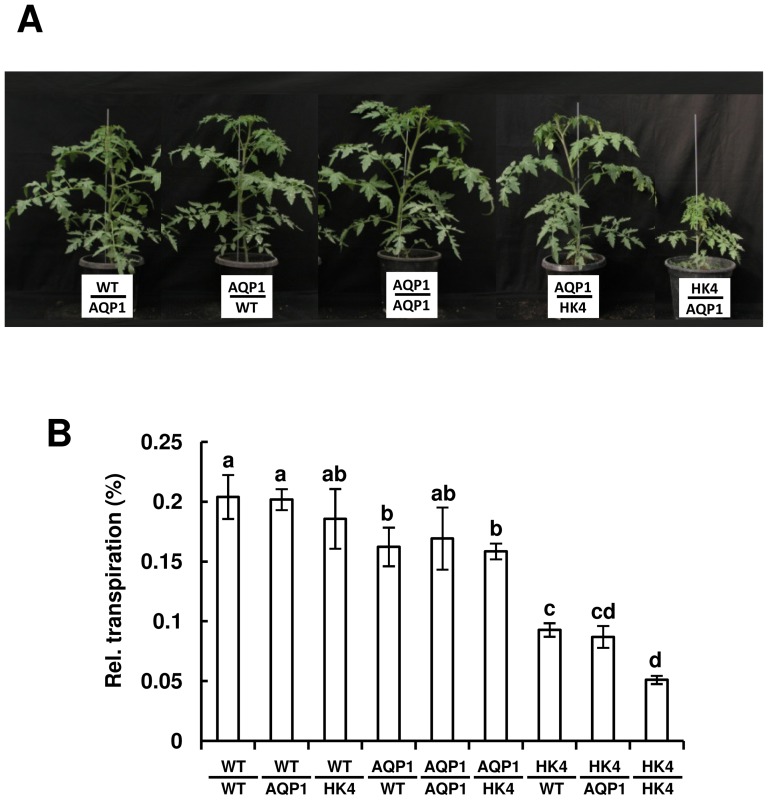
Reciprocal grafting and whole-plant relative daily transpiration. Reciprocal grafting of WT, AQP1 and HK4 plants was performed at the seedling stage. The plants were photographed (A and Fig. 2A in [30]) and their transpiration was measured about 4 weeks after grafting. (B) Whole-plant relative daily transpiration of reciprocal-grafted plants. Data were normalized to the total plant weight and the amount of water taken up by the neighboring submerged fixed-size wick each day, which was set to 100%. Presented data are means of four independent repeats ± SE. Different letters indicate a significant difference (*t* test, *P*<0.05).

To examine whether the decreased transpiration and growth imposed by *AtHXK1* and its complementation by *NtAQP1* emanate from distinct effects on roots or shoots, we created reciprocal grafts, in which WT, AQP1 and HK4 shoots were grafted onto WT, AQP1 and HK4 roots, covering all nine possible combinations. (Five combinations involving AQP1 are shown in Figure 5A and the other four combinations are shown in Figure 2A of [30]). *AtHXK1* inhibited growth only when expressed in shoots, independent of the root genotype (Fig. 5A and [30]). Similarly, measurements of cumulative whole-plant relative daily transpiration of the grafted plants indicated that *AtHXK1* decreased transpiration by about 50% only when expressed in shoots, independent of the root genotype [(Fig. 5B), in line with our recent discovery that *AtHXK1* stimulates stomatal closure and reduces transpiration when expressed in shoots [30].(i.e., *NtAQP1* in roots had no complementation effect on HK4 shoots) (Fig. 5A). These results show that separate expression of *NtAQP1* and *AtHXK1* in roots or shoots is insufficient to achieve complementation of *AtHXK1* phenotypes by *NtAQP1* and that the complementation of *AtHXK1*effects by *NtAQP1* occurs only when both genes are expressed simultaneously in the shoots.

### *NtAQP1* does not improve hydraulic conductance of *AtHXK1* plants, but does increase the conductance of CO_2_ in the leaf mesophyll and the rate of photosynthesis

The enhanced transpiration of the double-transgenic plants relative to HK4 plants might suggest that *NtAQP1* could potentially improve the low hydraulic properties of HK4 plants. We, therefore, measured the root and stem hydraulic conductivity of the WT, AQP1, HK4 and double-transgenic plants. *NtAQP1* did not improve the root conductance or xylem-specific stem hydraulic conductivity (*L*_r_ and *K*_sx_, respectively) of the double-transgenic plants, which remained low, as in the HK4 plants (Table 1). However, gas-exchange analysis of the double-transgenic plants revealed that *NtAQP1* increased photosynthesis rates (*A*_N_), CO_2_ conductance (*g*_m_) and stomatal conductance (*g*_s_), with no effect on intracellular CO_2_ concentration *(C*_i_), as compared to the low *A*_N_, *g*_s_ and *g*_m_ values observed in the HK4 plants (Table 1). In addition, *NtAQP1* increased both the concentration of CO_2_ in the chloroplasts (*C*_c_) and the electron transport rate (*J*), as compared to the HK4 plants (Table 1). We, therefore, suggest that the complementation of *AtHXK1* effects by *NtAQP1* is primarily due to the role of *NtAQP1* as a CO_2_ facilitator, which enhances the conductance of CO_2_ in the mesophyll thereby elevating the rate of photosynthesis despite the low expression of *CAB1* in AQP1xHK4 plants.

## Discussion

PIP1-AQPs were shown to enhance cell permeability to both CO_2_ and water [7], [13], [18]. Overexpression of *NtAQP1* in tobacco plants enhanced leaf mesophyll CO_2_ conductance (*g*_m_), hydraulic conductivity, stomatal conductance (*g*_s_), transpiration and photosynthesis (*A*_N_) [18], [20], [21]. Expression of *NtAQP1* in tomato plants also enhanced photosynthesis, stomatal conductance and transpiration [22]. However, in our study, *NtAQP1* did not enhance photosynthesis, stomatal conductance or hydraulic conductivity relative to WT plants (Fig. 5, Table 1) and enhanced transpiration only slightly (Fig. 4). These differences may be due to the different tomato genotype used in our study (MP1- [31], an indeterminate variety) or to different expression levels of *NtAQP1*. Nevertheless, photosynthesis, stomatal conductance and transpiration were elevated by *NtAQP1* in the double-transgenic plants (AQP1xHK4), as compared to the HK4 parental (isogenic) line. Yet, the hydraulic conductivity of AQP1xHK4 remained low as in the HK4 plants, implying that the increased transpiration that was observed is not directly related to hydraulic characteristics. Rather, the increased transpiration is most likely due to high *g*_m_ values in the mesophyll, which opens stomata and increases the influx of CO_2_ to help maintain constant levels of *C*_i_ in the substomatal cavity [44], [45]. High levels of *A*_N_, *g*_s_ and *g*_m_, accompanied by constant *C*_i_, were also reported in previous studies of tobacco plants overexpressing *NtAQP1* [18], [21], [42].

*AtHXK1* is a sugar-sensing enzyme that inhibits the expression of photosynthetic genes, decreases chlorophyll levels and reduces the rate of photosynthesis in response to increasing sugar levels [25]–[29]. As a result, tomato and Arabidopsis plants with high levels of *AtHXK1* expression display severe growth inhibition directly correlated to *AtHXK1* expression and activity levels [25], [26]. It is likely that part of the growth inhibition imposed by *AtHXK1* is the result of insufficient photosynthesis, since the increased photosynthesis rate observed in AQP1xHK4 plants partially eliminated this growth inhibition.

The increased rate of photosynthesis observed in AQP1xHK4 plants, despite the low level of expression of the photosynthetic gene *CAB1* in those plants, can probably be attributed to *NtAQP1*, which accelerates CO_2_ mesophyll conductance (*g*_m_) [21], [22]. The CO_2_ mesophyll conductance of HK4 plants is significantly lower than that of WT plants and is enhanced by simultaneous expression of *NtAQP1*, indicating that CO_2_ mesophyll conductance significantly affects growth.

It appears that, in addition to its known sugar-sensing effect (reducing expression of photosynthetic genes and reducing the rate of photosynthesis [25]–[27], [29], [46]; Fig. 2E and Table 1), *AtHXK1* also reduces *g*_m_, perhaps by reducing the expression of TRAMP (Fig. S2), the tomato homolog of *NtAQP1* [47]. Indeed, lower *g*_m_ levels have been observed in tobacco *NtAQP1* antisense lines [21] and Arabidopsis *pip1;2* mutants (a CO_2_-facilitating AQP;[24]). In those studies, the decrease in *g*_m_ was accompanied by lower *C*_c_. In agreement with the findings of those studies, the HK4 plants in our study exhibited lower *C*_c_ than the WT plants and the expression of *NtAQP1*in the double-transgenic plants (AQP1xHK4) led to full complementation of *C*_c_ (Table 1). Interestingly, the HK4 plants had lower electron transport rates (*J*) than the WT plants, while a clear recovery was observed in the AQP1xHK4 plants (Table 1) despite the low level of expression of the photosynthetic gene *CAB1* in the AQP1xHK4 plants (Fig. 2E).

It has previously been shown that expression level of *NtAQP1* which affects *g*_m_ levels also affects electron transport rates (*J*) [21], [24], [48]. Flexas et al. [21] hypothesized that modified intercellular CO_2_ concentrations may trigger differences in the leaf photosynthetic capacity, so that the photosynthetic machinery can adjust to the change in mesophyll conductance. This would also explain why *g*_m_ usually scales with photosynthetic capacity, as has been observed in broad comparisons of different species [49], [50].

The effect of *AtHXK1* on *g*_m_ suggests that HXK might coordinate photosynthesis with sugar levels by several mechanisms in different cell types. It inhibits expression of photosynthetic genes [25], [46] and reduces *g*_m_ most likely in mesophyll photosynthetic cells. In guard cells HXK mediates stomatal closure in response to sugars and reduces stomatal conductance (*g*_s_) [26], [30]. These findings support the existence of a multilevel feedback-inhibition mechanism that is mediated by HXK in response to sugars. When sugar levels are high, likely when the rate of photosynthesis exceeds the rate at which the sugar is loaded and carried by the phloem, the surplus of sugar is sensed by HXK in mesophyll and guard cells, which respond in concert to reduce both unnecessary investments in photosynthetic capacity and water loss. This response includes reducing the expression of photosynthetic genes, slowing chlorophyll production, diminishing mesophyll CO_2_ conductance and closing the stomata.

In addition to these effects in shoots, HXK reduces the hydraulic conductivity of stem and roots via an as yet unknown mechanism. This reduction in hydraulic conductivity occurs independently of stomatal conductance, as it also happens in the double-transgenic plants that have WT levels of stomatal conductance (Table 1). Nevertheless, grafting experiments indicate that neither overexpression of *AtHXK1* in roots nor expression of *AtHXK1* in the stem has any visible physiological effects. Rather, overexpression of *AtHXK1* in shoots is necessary and sufficient to obtain a photosynthesis effect and growth inhibition [25], [30]. The dominant effect of *AtHXK1*, lowering hydraulic conductance in AQP1xHK4, might be the reason for the intermediate transpiration rate of AQP1xHK4 plants, which is lower than that of WT plants (Fig. 4), despite the increase in stomatal conductance to levels similar to that of WT plants (Table 1). It has been suggested that NtAQP1 might play independent roles in leaves and roots, a hydraulic role in roots and a membrane CO_2_ permeability role in shoots [22]. The improved *g*_m_ observed in the double-transgenic plants supports the notion that, in leaves, NtAQP1 functions as a CO_2_ transmembrane facilitator and that the complementation effect of NtAQP1 may be primarily attributed to its affect on CO_2_ conductance in leaf mesophyll. The roles of HXK and PIP1 in the regulation of photosynthesis, stomatal conductance and transpiration are well established [18], [20]–[22], [25]–[29]. This study suggests that HXK and PIP1 *together* may influence these central properties of plant physiology and, eventually, plant growth.

## Supporting Information

Figure S1 **Expression analysis of** ***NtAQP1*** **in AQP1 transgenic line: Presence of NtAQP1 DNA, RNA and protein.** (A) The presence of *NtAQP1* was assayed by PCR using *NtAQP1*-specific primers; transgenic AQP1 plants yielded the expected 930-bp product. WT is a negative non-transformed wild-type plant. + stands for a positive PCR control with a plasmid containing *NtAQP1*. Ladder: 100-bp ladder. (B) cDNA of AQP1 was subjected to semi-quantitative PCR using *NtAQP1*-specific primers; Fwd-CCGGGCAGGTGTACTATCC, Rev-TGCCTGGTCTGTGTTGTAGAT. Amplification was performed using 35 PCR cycles. *SlCyP* (cyclophilin – accession; M55019) was used as a control. (C) Western blot analysis of protein extracts from AQP1 plants probed with *NtAQP1*-specific antibody (upper panel); Ponceau red staining of the Western blot indicating equal protein loading (lower panel). Western blot analysis and Ponceau staining were performed exactly as described in Sade et al. [22]).
(TIF)

Figure S2 **Expression of the TRAMP is suppressed by** ***AtHXK1*** **.** Expression level of TRAMP (tomato ripening associated membrane protein, accession no. NM_001247210), the tomato *NtAQP1* homolog, was determined by quantitative real-time PCR using cDNA extracted from leaves of WT and HK4 plants. Data are means of five independent biological repeats ± SE. Different letters indicate a significant difference (*t* test, *P*<0.05). *SlCyP* (cyclophilin) was used for normalization.
(TIF)
