# Supplementary figures and images for: Relationship between Hexokinase and the Aquaporin PIP1 in the Regulation of Photosynthesis and Plant Growth
Source: PLoS One. 2014 Feb 3;9(2):e87888. doi: 10.1371/journal.pone.0087888 (PMC3912149; doi:10.1371/journal.pone.0087888)

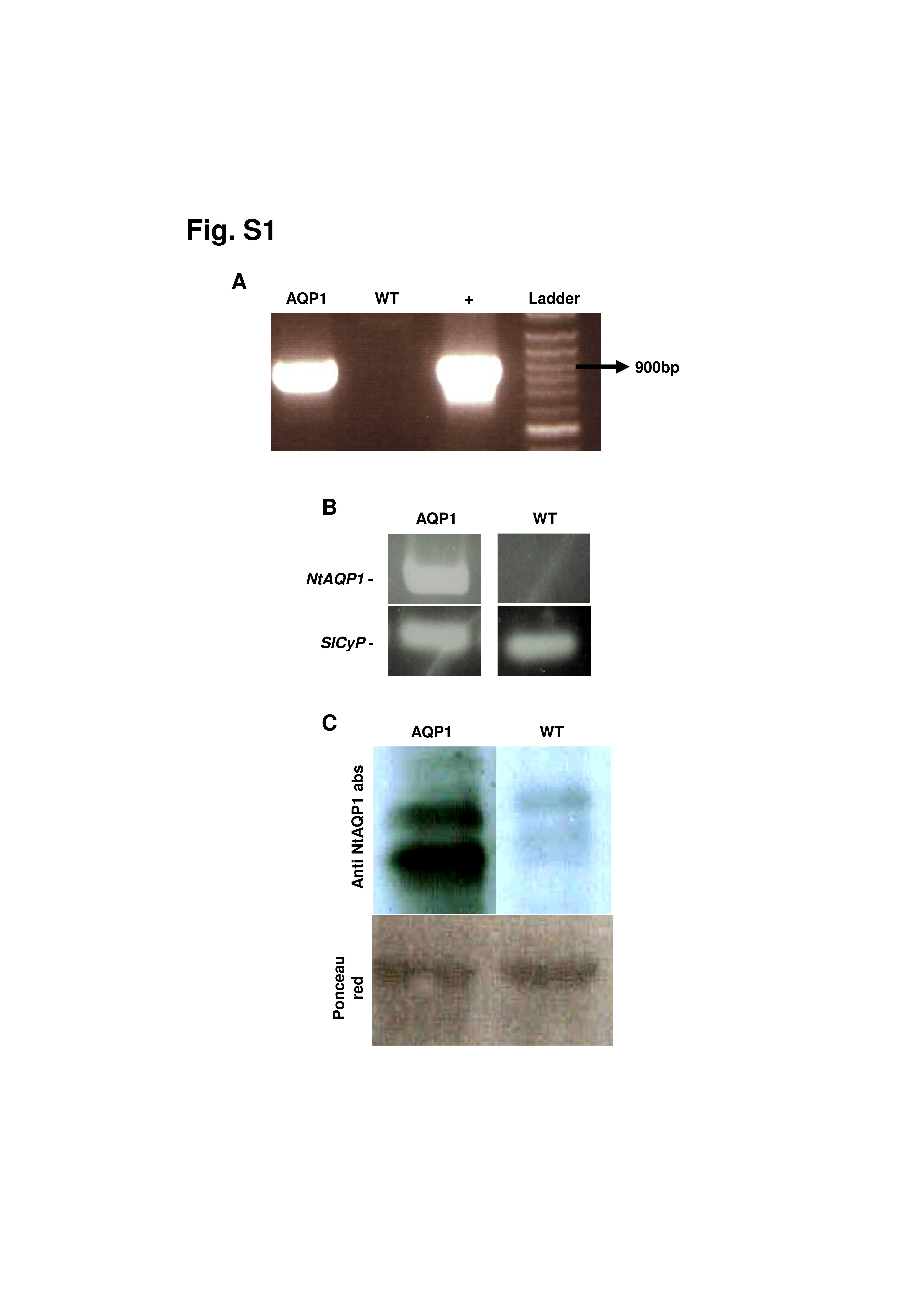

Supplement: Figure S1 — Expression analysis of NtAQP1 in AQP1 transgenic line: Presence of NtAQP1 DNA, RNA and protein. (A) The presence of NtAQP1 was assayed by PCR using NtAQP1-specific primers; transgenic AQP1 plants yielded the expected 930-bp product. WT is a negative non-transformed wild-type plant. + stands for a positive PCR control with a plasmid containing NtAQP1. Ladder: 100-bp ladder. (B) cDNA of AQP1 was subjected to semi-quantitative PCR using NtAQP1-specific primers; Fwd-CCGGGCAGGTGTACTATCC, Rev-TGCCTGGTCTGTGTTGTAGAT. Amplification was performed using 35 PCR cycles. SlCyP (cyclophilin – accession; M55019) was used as a control. (C) Western blot analysis of protein extracts from AQP1 plants probed with NtAQP1-specific antibody (upper panel); Ponceau red staining of the Western blot indicating equal protein loading (lower panel). Western blot analysis and Ponceau staining were performed exactly as described in Sade et al. [22]). (TIF) [file pone.0087888.s001.tif]

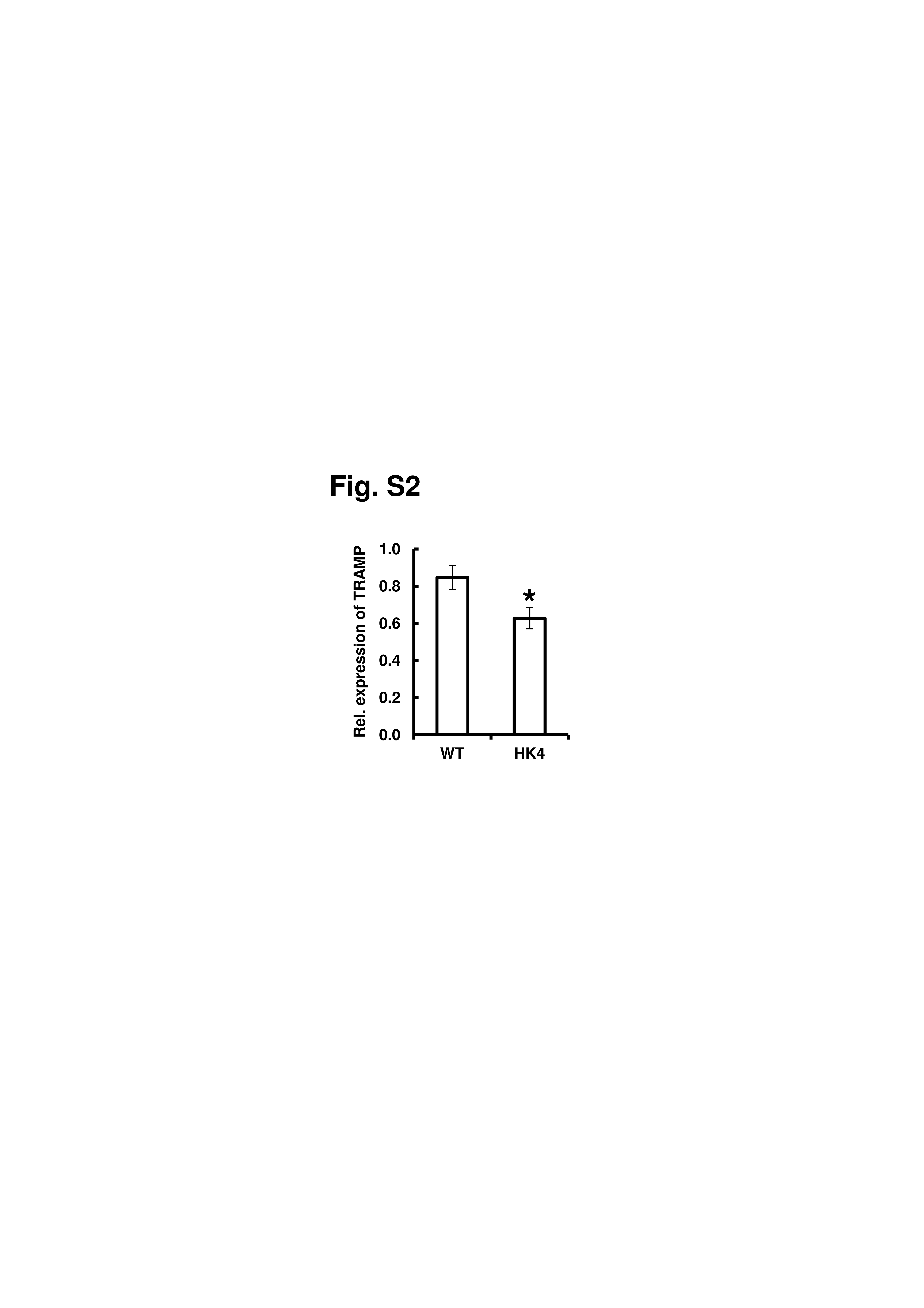

Supplement: Figure S2 — Expression of the TRAMP is suppressed by AtHXK1 . Expression level of TRAMP (tomato ripening associated membrane protein, accession no. NM_001247210), the tomato NtAQP1 homolog, was determined by quantitative real-time PCR using cDNA extracted from leaves of WT and HK4 plants. Data are means of five independent biological repeats ± SE. Different letters indicate a significant difference (t test, P<0.05). SlCyP (cyclophilin) was used for normalization. (TIF) [file pone.0087888.s002.tif]
